# Dynamic functional MRI markers of drowsiness during sleep onset period

**DOI:** 10.1016/j.isci.2025.113088

**Published:** 2025-07-10

**Authors:** Ivan Igor Gaez, Elpidio Attoh-Mensah, Clément Nathou, Lydie Vincent, Marc Joliot, Luc Brun, Mikaël Naveau, Olivier Etard

**Affiliations:** 1 Normandie Université, UNICAEN, INSERM, COMETE, CYCERON, CHU Caen, Caen, France; 2 Université de Limoges, HAVAE, UR 20217, 87000 Limoges, France; 3 GIN, UMR5293, CEA, CNRS, Université de Bordeaux, Bordeaux, France; 4 Normandie Université, UNICAEN, ENSICAEN, CNRS, GREYC, 14000 Caen, France; 5 Normandie Université, UNICAEN, CNRS, UMS3408 CYCERON, Caen, France

**Keywords:** Health sciences, Neuroscience

## Abstract

The present use of ocular activity to enhance the temporal resolution of the classification of sleep onset period (SOP) allowed to reveal an increase in the power of oscillations in the BOLD signal at 0.05 Hz in the brain, particularly in sensory cortices, as soon as the first signs of drowsiness. These changes were evident in the “likely drowsy” state of SOP, representing early sleep-related vigilance fluctuations, often encountered even during activities like driving. Our findings show changes in the BOLD signal even before conventional indicators of sleep onset.

## Highlights

• BOLD oscillation at 0.05 Hz rises with drowsiness during sleep onset period (SOP)
• Drowsiness detected via eyelid activity shows early changes in brain oscillations
• PSD_0.05_ increase found in 58 brain regions, mainly sensory, visual, and motor areas

## Graphical abstract

**Figure:**
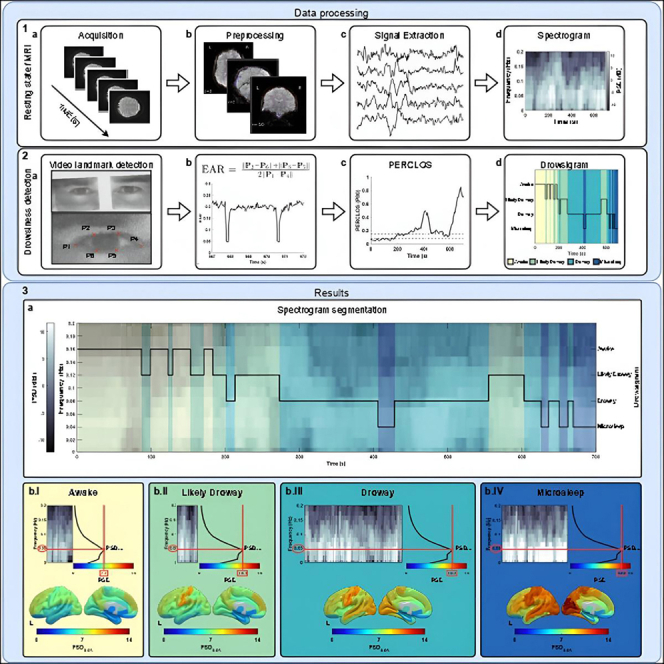

## Introduction

Over time, our understanding of human sleep cycles has evolved through the meticulous observation and analysis of brain activity. Polysomnography (PSG), the gold standard technique in sleep measurement, incorporates the capture of scalp electroencephalographic (EEG) recordings to comprehensively assess and monitor sleep patterns.^1^ The American Academy of Sleep Medicine (AASM) and its standard scoring system consider various sleep stages, ranging from light (N1 and N2) to deep sleep (N3), by unraveling the electrical activity of neurons.^1^ While the transition from light to deep sleep is well-documented, featuring EEG graphoelements such as K-complexes (KC) or sleep spindles, it is more challenging to characterize the sleep onset period (SOP).^2^

The SOP encompasses a series of intermediary states that alternate between wakefulness and sleep.^2^^,^^3^^,^^4^^,^^5^ Consequently, SOP and particularly its drowsiness states plays a pivotal role in the sleep process marking the separation of the individual from the surrounding environment.^2^^,^^5^ It also involves a gradual disengagement of the cortical regions, coupled with a reduction in the processing of sensory information.^6^^,^^7^ Thus, classifying this SOP poses challenges within the standard AASM framework, as its drowsiness states might be categorized variably as wakefulness or N1 contingent upon the expertise of the scorer.^2^^,^^5^^,^^8^^,^^9^ Indeed, the distinction between wakefulness and N1 stages primarily relies on analyzing the distribution of alpha rhythm (8–12 Hz) within a 30-s epoch, with the threshold set at 15 s of alpha rhythm (below corresponding to N1 and above to wakefulness).^1^ Therefore, this approach frequently leads to the misclassification of drowsiness, particularly because it can occur within shorter periods than 15 s.^2^^,^^5^^,^^10^ While these disparities have minimal influence on the overall results of full-night sleep analysis, they pose a significant constraint while studying the SOP.

The development of other non-invasive neuroimaging techniques in recent decades allowed to study in more detail brain activity associated with the different sleep stages.^7^^,^^11^^,^^12^^,^^13^^,^^14^^,^^15^^,^^16^ The functional magnetic resonance imaging (fMRI) and its blood-oxygen-level-dependent (BOLD) signal, have been thoroughly applied to complement EEG findings. It is widely demonstrated that brain activity decreases during sleep in most of the subcortical (brainstem, thalamus, basal ganglia, and basal forebrain) and cortical (prefrontal cortex, anterior cingulate cortex, and precuneus) regions.^17^ Interestingly, using resting-state fMRI (rs-fMRI), reports have indicated that this global reduction in brain activity would be associated with widespread changes in the whole brain functional connectivity.^7^ Recent studies have emphasized the presence of specific transitions and dynamics of brain connectivity during wakefulness, as well as N2 and N3 sleep stages.^14^ However, these transitions are not as evident during the N1 stage.^14^ This observation supports the prevailing consensus that the N1 sleep stage, as defined by AASM and EEG recordings, lacks clear delineation, partly due to inconsistencies introduced by SOP and its resulting drowsiness events.^18^ Examining local brain activity could potentially reveal distinct markers of SOP, as it is now understood that sleep can substantially modify local neuronal states.^15^ Some authors have utilized low-frequency (<0.1 Hz) oscillations of BOLD activity in brain regions and networks to investigate these localized sleep effects. Thus, an augmented low-frequency BOLD oscillation in cortical areas (frontal, posterior) and also in the thalamus and ventricles was found during sleep.^15^^,^^19^^,^^20^ Nevertheless, to make significant strides in our comprehension, it is crucial to refine the temporal categorization of SOP within the wake-N1 transition, beyond the classification set forth by the AASM.

Existing scoring systems such as Hori et al., enhance the identification of SOP by segmenting AASM stages into shorter intervals, thus improving the detection of isolated events.^21^^,^^22^ However, this approach may still lack comprehensiveness in capturing the dynamic nature of SOP and all its intermediate states, particularly concerning drowsiness.^2^ Indeed, drowsiness results from a high homeostatic pressure in favor of sleep and a low circadian pressure in favor of wakefulness, with various changes in the brain and body.^23^ This would suggest that in addition to EEG, numerous parameters such as cardiorespiratory or behavioral can be used as indicators of drowsiness.^23^ Reports suggested that EEG might not be the optimal parameter, as it primarily captures the synchronized activity of neuron populations but offers limited insight into the distinct reduced motor activity characteristic of drowsiness.^23^ Besides, the diversity of algorithms used to detect drowsiness from EEG prevents the comparison of different results.^24^

These observations highlight the necessity of developing a more widely accepted approach that also provides an improved classification of SOP. Quite interestingly, methodologies including various measures of psychomotor behavior such as ocular activity have been suggested to improve the detection of drowsiness,^25^ which could in turn enhance the classification of SOP.

Studies utilizing eyelids closures as a key measure have identified instances during SOP, where participants experienced low arousal state^26^^,^^27^ or even microsleep.^28^ However, SOP is a gradual process that encompasses multiple transitional and alternating states between wakefulness and sleep.^2^ Thus, investigating SOP in a more dynamic and nuanced way could provide valuable insights into the earliest indicators of SOP, as well as the various stages of transition that occur during this period before sleep onset. Since drowsiness is a key indicator, we believe that examining the neural correlates of its distinct stages, marked by eyelids closure, could allow to gain deep insights into the underlying mechanisms of SOP.

In the present study, we aimed to investigate brain activity related to drowsiness states during SOP, utilizing low-frequency BOLD oscillations from rs-fMRI. To consider the multilevel nature of SOP, we employed the classification based on the technique of the PERCLOS index, which assesses the percentage of eyelids closures over time to detect the different drowsiness states.^29^ This method commonly used in the field of road accident prevention,^30^ also eliminates the necessity for EEG settings that are complex and uncomfortable in an MRI environment.

## Results

Forty-one participants aged 18 to 36 (mean = 22.95 ± 4.54) were included in this study with an approximately 1:1 sex ratio (20 female; 21 male). The mean body mass index value (19.66 ± 2.17) was within normative ranges given the population age, and participants scored an average of 52.41 ± 7.99 at the circadian typology questionnaire.

### Drowsiness changes throughout resting state

**Figure 1:**
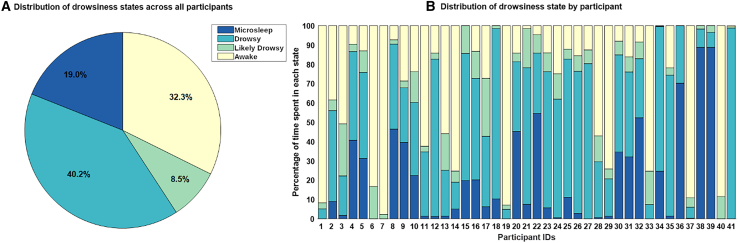
Distribution of the percentage of time spent in each drowsiness state (A) Percentage of overall time spent by all participants in each drowsiness state. (B) Percentage of time spent by each participant in the drowsiness states.

We will use the portmanteau word “drowsigram” to describe the drowsiness states. The “drowsigram” was defined through automatic image processing of the video captured by the MRI surveillance camera, suggesting 4 drowsiness states (awake, likely drowsy, drowsy, sleep, see supplemental information for details). We found that during the 45-min rs-fMRI scans, every participant attained at least the “likely drowsy” state, with 40 entering a drowsy state and 35 ending up falling asleep. A pie chart depicting the distribution of the percentage of time spent in each state of the “drowsigram” is presented in Figure 1. In addition, participants cycled through the states of the drowsigram multiple times, with less than one minute of uninterrupted microsleep on average (see Figures S3 and S4). Overall, our findings indicate that participants mostly (67% of the time) remained in a state other than awake. Furthermore the “drowsigram” aligns with the self-reported answers in the Resting State Questionnaire (RSQ). Indeed, after the examination, participants (21.95%) who reported not having slept while responding to the RSQ, spent more time in the awake state rather than in the other states of the “drowsigram” as outlined by the main effect of the ANOVA analysis (F_3,38_ = 14.288, *p* < 0.001).

### Low-frequency oscillations in BOLD signal

#### Specificity in frequency changes

**Figure 2:**
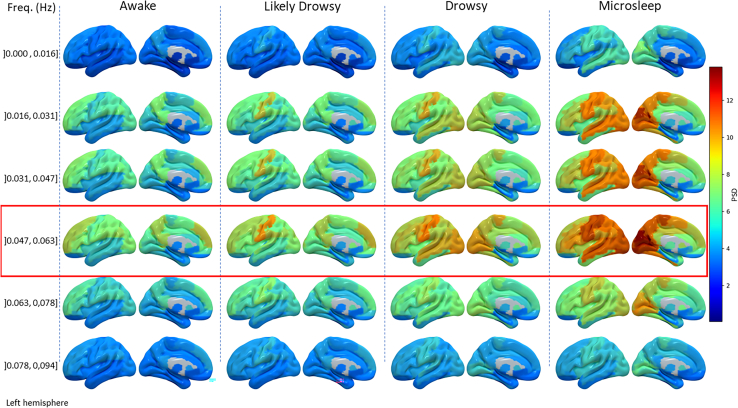
Surface rendering of power spectral density (PSD) of BOLD oscillations within region of interests in the atlas AAL3 Variations in PSD, across frequency bands ranging from 0 to 0.1 Hz (rows) and across drowsiness states (columns), are depicted. The red box highlights the frequency band with the highest PSD. Each drowsiness state column contains two sub-columns. The sub-columns represent left-brain hemisphere with the outer and inner faces at the right and left sides of the figure, respectively.

A surface rendering illustrating the power spectral density (PSD) of BOLD oscillation in the 6 sleep-related frequency bands, is presented in Figure 2. We observed a progressive increase in PSD throughout the frequency bands, reaching its peak within the range of ]0.047, 0.063], specifically at the value of 0.05 Hz. Following this, the power decreases while approaching 0.1 Hz. This phenomenon is consistent across all states of the “drowsigram” and is observable throughout the whole brain. We identified a positive correlation between PSD0.05 and PERCLOS in all ROIs. The strongest correlations were observed in primary sensory cortices (r^2^ > 0.15 see Figures S5–S9). Despite being statistically significant, these correlations were moderate or even low in the deep structures such as the thalamus (r^2^ = 0.03 to 0.09). To delve deeper into these findings, the subsequent analyses exclusively focus on the PSD of oscillations at 0.05 Hz (PSD_0.05_).

#### Changes in power spectral density within regions of interests across drowsigram

**Figure 3:**
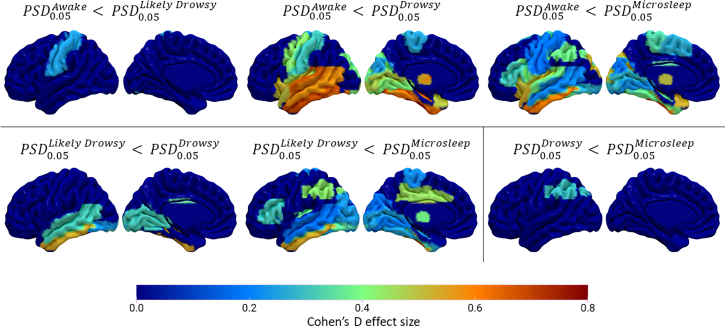
Surface rendering of the results from post-hoc comparisons of the power spectral density of BOLD signal oscillations at 0.05 Hz (PSD0.05), between different drowsiness states within each ROI in the AAL3 atlas Effect sizes, calculated using Cohen’s d, are depicted in regions where the difference between two states was statistically significant after Bonferroni correction (*p* < 0.00005, as 984 comparisons were computed). Outer and inner faces of the left-brain hemisphere are represented.

The generalized linear models (GLMs) computed across the AAL3 1 mm atlas and corrected for multiple comparisons using the Bonferroni method (*p* < 0.0003, see supplemental information for details), allowed to demonstrate a significant variation of PSD_0.05_ across up to 58 ROIs (Table 1), as a function of the “drowsigram” (Wald’s χ^2^ ranging from 15.90 to 111.41, see Tables S1 and S2). Moreover, post hoc analyses, also corrected with the Bonferroni method (*p* < 0.00005 as we computed 984 pairwise comparisons), allow us to specify the different states of the “drowsigram” that differed within the regions (Figure 3). Thus, the PSD_0.05_ gradually increases while transitioning from wake to microsleep. During this progression, we first identified the PSD_0.05_ increase in the postcentral cortex and cerebellum (awake < likely drowsy state; mean Cohen’s d = 0.27), then in the primary visual and temporal cortices (likely drowsy < drowsy; mean Cohen’s d = 0.35) and in the inferior parietal region (drowsy < sleep; Cohen’s d = 0.31). Furthermore, as displayed in Figure 3, we also identified this difference in PSD_0.05_ in several other regions in the other pairwise comparisons across the “drowsigram” (see Tables S1 and S2 for details). The most marked differences occurred between awake and microsleep states in sensory cortices and deep structures such as the thalamus (mean Cohen’s d = 0.36).

**Table 1:** Overview of statistically significant changes in PSD_0.05_ across the drowsigram

Pairwise comparisons		ROI^a^	Networks^b^
Awake	Likely Drowsy	2	Somatomotor
	Drowsy	52	Visual, Somatomotor, Limbic
	Microsleep	58	Visual, Somatomotor, Frontoparietal
Likely Drowsy	Drowsy	11	Visual
	Microsleep	34	Visual
Drowsy	Microsleep	1	Visual

PSD_0.05_: Power Spectral Density of of BOLD oscillation at 0.05 Hz. Analyses corresponded to Bonferroni-corrected pairwise comparisons after generalized linear models for the 164 ROIs in the AAL3 Atlas (*p* < 0.00005) or the linear mixed model for the networks in the atlas of Thomas Yeo et al.

a Count of statistically significant AAL3 atlas Regions of Interest (ROIs).

b Statistically significant networks in the atlas of Thomas Yeo.

#### Changes in power spectral density within resting-state networks across drowsigram

**Figure 4:**
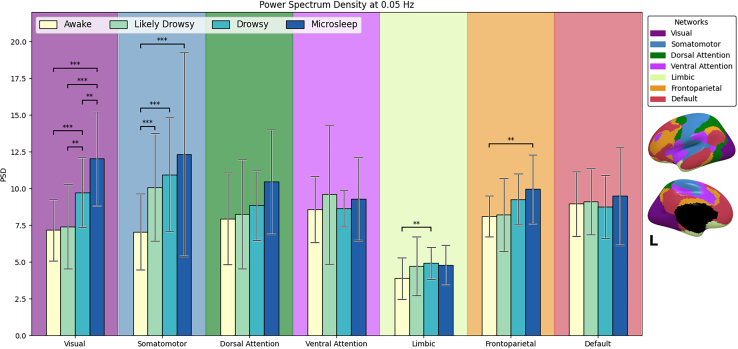
Power spectral density of the oscillations of BOLD signal at 0.05 Hz (PSD_0.05_), for each network in the YEO atlas Bar chart depicting PSD_0.05_ of each drowsiness state within each network. Data are presented as mean ± SEM. Networks are identified by colors illustrated in the legend at the top right, as well as in the surface rendering below it, and drowsiness states by colors depicted in the legend at the top left corner within the bar chart. ∗*p* < 0.05; ∗∗*p* < 0.01 and ∗∗∗*p* < 0.001 for Bonferroni-corrected pairwise comparisons after a linear mixed model.

Figure 4 and Table 1 detail the evolution of PSD0.05 within each network of the atlas of Thomas Yeo et al. A linear mixed model revealed an effect of the states of the “drowsigram” (F_3,961.07_ = 33.49 *p* < 0.001) and of the network (F_6,961.07_ = 60.39 *p* < 0.001) on the PSD_0.05_.

Given that we also found an interaction effect (F_18,961.07_ = 4.7, *p* < 0.001), we computed post hoc pairwise comparisons of the different states of the “drowsigram” within each network with Bonferroni corrections (*p* < 0.001 as we computed 42 pairwise comparisons). This showed that the PSD_0.05_ significantly changed across the “drowsigram” within up to four of the seven networks (Table 1). These changes were noticeable within the visual network for all comparisons (mean Cohen’s d = 0.30) except awake vs. likely drowsy. Regarding the somatomotor network, the awake state was different from all the other states (mean Cohen’s d = 0.19). Conversely, there were no significant changes of PSD_0.05_ within the dorsal attention, ventral attention, and default mode network (DMN).

## Discussion

The present investigation sheds new light on the dynamics of brain activity during sleep onset. We discovered that the earliest manifestations of drowsiness during SOP detected by the dynamic of eyelids closures, coincide with an increase in the power spectral density of BOLD oscillations at the frequency of 0.05 Hz. These changes became evident upon participants entering the “likely drowsy” state of the “drowsigram,” with the most marked differences between awake and microsleep states. Crucially, our findings underscore a specific spatial distribution of such BOLD oscillations within brain regions relevant to somatomotor and visual functions.

The current findings are consistent with previous studies indicating that the BOLD signal may fluctuate during rest periods, potentially indicative of sleep onset.^15^^,^^31^^,^^32^^,^^33^^,^^34^ The work of Fukunaga et al. in 2006 was one of the first to demonstrate that the evolution of the BOLD signal during extended rest periods, leads to large amplitude variations that authors associated with sleep events. While most of these studies considered sleep onset within an AASM framework, our study offers valuable insights by introducing a more temporally refined classification with the dynamic of eyelids closures. This is particularly interesting as prior efforts to use eyelids closures have primarily focused on neural correlates of instances of low arousal or microsleep^26^^,^^28^ rather than providing a comprehensive understanding of the dynamic progression of drowsiness during SOP. Indeed, we identified an increase in PSD of low-frequency BOLD oscillations during the transition from wake to microsleep with the emergence of changes as early as the “likely drowsy” state. This is a significant discovery as the “likely drowsy” state of the “drowsigram” represents one of the earliest manifestations of sleep-related vigilance fluctuations, often encountered even during activities like driving.^30^ We were thus able to showcase changes in the BOLD signal well before conventional indicators of sleep onset. This corroborates the well-known lack of clear-cut delineation of N1, which is the early known sleep stage, partly because of the misclassification of drowsiness during SOP.^2^^,^^18^

Subsequently, the increase in PSD of low-frequency BOLD oscillations across the “drowsigram,” peaked at 0.05 Hz which is at first a valuable argument in favor of the relevance of the rs-fMRI technique to improve knowledge on the SOP. Indeed, in sleep studies leveraging EEG and AASM framework, the lowest frequency of oscillation of brain electrical activity (Delta activities: 0.5 Hz^3^^,^^18^; is ten times higher than the frequency (0.05 Hz) of BOLD oscillations in the present study. Notably, BOLD oscillations at 0.05 Hz closely match the cyclic alternating patterns (CAPs) identified during sleep as an indicator of sleep instability caused by internal and external factors.^35^^,^^36^ Interestingly, our participants were sleep-deprived and were specifically instructed to avoid falling asleep. Thus, sleep onset under such conditions, especially in the disruptive environment of a noisy MRI, would likely be precarious favoring the occurrence of CAPs.^35^^,^^36^ Therefore, it could be assumed that CAPs were among the physiological modifications underlying this low-frequency BOLD oscillation during SOP.

The increase in PSD_0.05_ during SOP was specific to some brain regions. In the ROI-wise analysis, the power increased progressively throughout the “drowsigram” first in the postcentral and cerebellum (awake vs. likely drowsy), then on occipital frontal, and temporal regions (likely drowsy vs. drowsy), and finally in parietal regions (sleep vs. drowsy). In addition, this progressive increase in PSD_0.05_ across the “drowsigram” states, was replicated in brain regions relevant to visual and somatomotor functions. This latter observation supports the notion that the BOLD oscillation at 0.05 Hz may have a sensory origin. This is also supported by prior studies, which similarly suggest a sensory explanation for other low-frequency BOLD oscillations observed during sleep.^15^ Henceforth, we hypothesize that the BOLD oscillation at 0.05 Hz could be the manifestation of the reduction of sensory processing to favor sleep, particularly during moments with high sleep pressure. This sensory hypothesis gains further traction when considered alongside rs-fMRI studies that include sleep graphoelements like KCs. Indeed, reports have indicated that when a KC is triggered by external stimuli, it results in an augmented BOLD signal within primary sensorimotor cortical areas.^33^^,^^34^ Interestingly, KCs are also acknowledged as a crucial component in the sleep continuum, bridging the gap between vertex waves in light sleep and the slow wave cycle in deep sleep.^37^ Therefore, it would not be surprising if KCs share the same neural activity background with the SOP as both play crucial roles in the downward progression of the sleep continuum, despite the latter occurring very early.

During SOP, it is noteworthy we found an absence of the BOLD signal oscillations at 0.05 Hz in brain regions of the DMN or attention networks. This finding might seem odd, considering numerous studies have highlighted significant changes within the DMN during sleep.^7^^,^^31^^,^^38^ However, the absence of DMN involvement could be attributed to several factors. Firstly, it could suggest that BOLD oscillations at 0.05 Hz might be specific to sensory networks, thereby reinforcing the validity of the sensory hypothesis to explain this variation during SOP. Secondly, since we are examining SOP and the earliest stages of sleep, regions in the DMN or attention networks may only show changes in oscillations at 0.05 Hz once sleep is fully established.

This work offers valuable insights into brain activity during the critical period of sleep onset detected by the innovative “drowsigram”. We found SOP-related oscillations in the BOLD signal at 0.05 Hz which occur in the sensory cortices in particular, as soon as the earliest manifestation of drowsiness. The dynamic and progressive nature of SOP is thus reflected in brain activity, and tracking drowsiness can provide valuable insight into the neural processes. An important contribution of this research lies in linking the BOLD signal to different moments of SOP achieved through the use of the eye closure dynamic. Indeed, SOP-related drowsiness is a known confounding factor in MRI data, and the present results highlight the value of using ocular activity via camera surveillance to improve the temporal precision of the detection of such SOP-related drowsiness. This approach is particularly relevant given the challenges of using EEG in MRI environments and the lack of temporal resolution of conventional EEG-based sleep scoring systems. Future investigations should build upon the “drowsigram” framework to delve deeper into the relationship between BOLD signal and drowsiness, refine existing correction methods, and enhance the rs-fMRI signal quality.

### Limitations of the study

The main limitation of the present study would be the light sleep deprivation applied the night before the experiments. This could have led to the process of falling asleep not following its usual course. However, we applied mild sleep deprivation to facilitate sleep onset and ensure the collection of a sufficient amount of data. Furthermore, people spent approximately 20% of the duration of this experiment in a microsleep state which is similar to previous studies of sleep in an MRI environment.^39^^,^^40^ Furthermore, questions may arise regarding how the drowsigram presented in this study corresponds with simultaneous EEG recordings. Interestingly, the PERCLOS index used for this classification is validated regarding its ability to detect drowsiness changes^30^^,^^41^ and most importantly, it matches the states of drowsiness and sleep reported by our participants. In addition, a recent study demonstrates that EEG data can be used in an automatic classification to predict the PERCLOS index.^41^

## Resource availability

### Lead contact

Further information and requests for resources and reagents should be directed to and will be fulfilled by the lead contact, Olivier Etard (olivier.etard@unicaen.fr).

### Materials availability

This study did not generate new unique reagents.

### Data and code availability

• All data supporting the findings of this study are available from the corresponding author upon reasonable request.
• The code used for preprocessing and analysis is available upon request.
• Any additional information required to reanalyze the data reported in this paper is available from the lead contact upon request.

## Acknowledgments

This work was co-funded by the Normandy County Council, the 10.13039/501100000780European Union (PredicAlert European Project - FEDER fund), and the 10.13039/501100001665French National Research Agency (ANR-11-INBS-0006 & ANR-24-CE19-4467).

## Author contributions

Conceptualization, I.I.G., E.A.-M., C.N., M.N., and O.E.; methodology, I.I.G., E.A.-M., L.B., M.N., and O.E.; investigation, I.I.G., E.A.-M., C.N., L.V., and O.E.; data curation I.I.G., E.A.-M., M.J., L.B., M.N., and O.E., writing – original draft, I.I.G., E.A.-M., and O.E.; writing – review and editing, I.I.G., E.A.-M., L.V., C.N., L.B., M.J., M.N., and O.E.; funding acquisition, O.E.; resources, I.I.G., E.A.-M., and O.E.; supervision, O.E. and E.A.-M.

## Declaration of interests

The authors declare no competing interests.

## STAR★Methods

### Key resources table

**Table:** 

REAGENT or RESOURCE	SOURCE	IDENTIFIER
**Software and algorithms**		

fmriprep (v20.2.6)	Esteban et al. 2019^42^	https://doi.org/10.1038/s41592-018-0235-4
Nilearn	Abraham et al. 2014^43^	https://doi.org/10.3389/fninf.2014.00014
Dlib v19.24.1 (Python v3.10.13)	King, 2009^44^	http://dlib.net
IBM SPSS Statistics v24	IBM Corp.	https://www.ibm.com/products/spss-statistics

### Experimental model and study participant details

Young and healthy participants were included in this study if their age fell between 18 and 40 and had no contraindications for MRI examination. The study included an equal number of female and male participants and did not differentiate by race or ethnicity, although the sample was predominantly Caucasian (96%), with smaller representations of Black (3%) and other racial groups (1%). A physician assessed the non-inclusion criteria which were the following: neurological, cardiorespiratory or psychiatric disorders; being pregnant; long-term medical treatment and/or regular use of psychotropic drugs or other medication known to impair alertness or sleep; extreme profiles considering the Horne & Ostberg circadian typology questionnaire^45^; nighttime or night shift workers. This study was approved by the National Ethics Committee (no. 2020-A02858-31) and all included participants provided written informed consent to take part in the protocol.

### Method details

#### Experimental protocol

One week before the MRI acquisitions, participants were given a sleep diary to write down their bedtimes and wake-up times to control their sleep habits. To facilitate drowsiness during the rs-fMRI, participants were asked to follow a light sleep deprivation program (going to bed at 1:00 a.m. and getting up at 6:00 a.m.), the night before the exam. To prevent the effects of sleep homeostasis, the delay between awakening the day of the experiment and the rs-fMRI session was identical for all participants. To minimize individual differences in circadian rhythms, participants with extreme chronotypes were excluded based on the Horne and Ostberg questionnaire. The MRI acquisitions began with technical and anatomical sequences lasting around 15 minutes, followed by a rs-fMRI scan of 45 minutes. Participants were given the following rest instructions during rs-fMRI: “Remain laid down, be careful not to move, let your mind wander without engaging in structured mental tasks such as reciting a text or singing a song, keep your eyes opened but you are allowed to blink and try not to fall asleep”. No visual stimuli, such as a fixation cross or point, were presented during the experiment. The ambient light from the MRI tube's integrated source was dimmed to its minimum level and remained on for the entire duration of the acquisition. Concurrently, the subject’s eyes were recorded via MRI’s video surveillance camera (WAT-600CX, Watec Co., Japan). At the end of the session, a RSQ^46^ was filled out by the participants.

#### MRI acquisitions

We conducted anatomical and resting-state brain imaging using a 3T MRI scanner (MRI GE 3T SIGNA Premier). For anatomical data acquisition, we employed a high-resolution T1-weighted (T1w) via the MPRAGE sequence (TR = 2.3 s, TE = 3 ms, flip angle = 8°, spatial resolution = 1x1x1 mm^3^). Subsequently, we applied a 45-minute T2∗ gradient echo sequence during the rs-fMRI scan (TR = 1 s, TE = 30 ms, flip angle = 62°, slice thickness = 2.4 mm, spatial resolution 2.4x2.4 mm^2^, Hyperband acceleration = 6, 70 slices, 2700 volumes) (see graphical abstract).

#### MRI data preprocessing

Preprocessing of rs-fMRI data was performed using fmriprep software (v20.2.6).^42^ T1w MRI images of all participants underwent a first correction for intensity and non-uniformity to remove intensity variations on the image caused by scanner artifacts. Subsequently, the images were skull striped, followed by the brain tissue segmentation into the cerebrospinal fluid, white-matter and gray-matter. Finally, we applied a spatial normalization to the MNI space. Preprocessing of the functional data, began with the creation of a skull-stripped reference volume. This volume underwent distortion correction and was then aligned to the T1w anatomical reference. Subsequently, we performed a head motion parameter estimation and a slice-timing correction. fMRI time-series were resampled into their original space and standard MNI space.

#### BOLD time-series extraction from rs-fMRI data

The time-series for each rsfMRI scan were extracted using the Nilearn Python library.^43^ A total of 164 signals were extracted from each participant's scan using the AAL3 1 mm atlas.^47^ This extraction process involved a careful consideration of various factors, including the preprocessed BOLD signal, the anatomical mask of each participant in MNI125 space, and a set of confounds. We chose the confounds according to anatomical and functional data preprocessing steps and they comprise the following: 24 head motion parameters (consisting of six base motion parameters, their six temporal derivatives, and 12 quadratic terms derived from the initial parameters and their temporal derivatives), discrete cosine-basis regressors (equivalent to using a high-pass filter with a cut-off frequency of 0.01 Hz), as well as measures of Global Signal (GS), White Matter (WM), Cerebrospinal Fluid (CSF), and a combined measure of WM and CSF (see graphical abstract). This approach was taken to mitigate the physiological effect on the rs-fMRI output,^40^ and is considered to result in a clear BOLD signal.^48^

#### Drowsiness assessment

We will use the portmanteau word “drowsigram” to describe the different drowsiness states. The “drowsigram” was defined through an automatic image processing of the video captured by the MRI surveillance camera, suggesting 4 drowsiness states (awake, likely drowsy, drowsy, microsleep). We used an eye landmark detection algorithm35 to automatically label eye contours, followed by a manual inspection and correction of these annotations (see Figures S1 and S2). The eye landmark detection model was trained using Dlib’s (dlib v19.24.1, python v3.10.13)^44^ implementation of Kazemi’s 1-ms face alignment algorithm which consists of a set of cascaded regression trees.^48^ Error metrics were computed using the dedicated Dlib function for this purpose, which is based on the average pixel distance between the ground truth and predicted landmarks using the following equation:

(Equation 1) $$Error=\frac{1}{N}\sum_{i=1}^{N}{\left\|{\hat{p}}_{i}-p_{i}\right\|}_{2}$$

Where ${\hat{p}}_{i}$ is the predicted 2D coordinate of the $i^{th}$ landmark, $p_{i}$ is the corresponding ground truth coordinate and N is the total number of landmarks and ${\left\|\cdot \right\|}_{2}$ the Euclidean distance between. ${\hat{p}}_{i}$ and $p_{i}$ (L2-norm).

The computed metrics gave us an error of 3.0 pixels for the training dataset and an error of 13.4 pixels for the test part.

Next, the landmarks were used to compute the Eye Aspect Ratio (EAR) of the left eye, following the method proposed by Soukupová and Čech (2016),^49^ using the following formula:

(Equation 2) $$EAR=\frac{\left\|p_{2}-p_{6}\right\|+\left\|p_{3}-p_{5}\right\|}{2\left\|p_{1}-p_{4}\right\|}$$

Where $p_{1}$ through $p_{6}$ are the landmark points detected by the algorithm.

We determined the EAR on a frame-by-frame basis, yielding a signal that captured the eye blinks of the subjects over time. Thus, the lower the EAR, the more closed the eyes are, and the higher the EAR, the more open the eye. The maximum and minimum EAR values were subject-dependent. Once the EAR had been calculated for each participant, a supervised process was conducted to rectify errors in the detected landmarks on their faces, using an in-house MATLAB user interface (see Figure S2).

After artefact removal, we normalized each participant’s EAR signal to the range [0, 1] as follows:

(Equation 3) $${E\tilde{A}R}_{s}\left(t\right)=\frac{{EAR}_{s}\left(t\right)-\min_{b}\left({EAR}_{s,b}^{\min}\right)}{\max_{b}\left({EAR}_{s,b}^{\max}\right)-\min_{b}\left({EAR}_{s,b}^{\min}\right)}$$

Where t denotes time (or frame index) and ${EAR}_{s,b}^{\min}$ and ${EAR}_{s,b}^{\max}$ represent the minimum and maximum EAR values within blink b of participant s, respectively.

After this rescaling process, a binarization of ${E\tilde{A}R}_{s}\left(t\right)$ was performed to facilitate the computation of the PERCLOS index, which is the percentage of time a person’s eyes are at least 80% closed within a sixty-second window.^29^ This process is formalized as follows:

(Equation 4) $$C_{s}\left(t\right)=\left\{ \begin{array}{c} \;1,E{\tilde{A}R}_{s}\left(t\right)\leq 0.8\\ 0,{E\tilde{A}R}_{s}\left(t\right)>0.8 \end{array}\right.$$

(Equation 5) $$\text{PERCLO}S_{s,k}=\frac{1}{N_{60}}\sum_{n=-\lfloor N_{60}/2\rfloor }^{\left\lfloor \left(N_{60}-1\right)/2\right\rfloor }C_{s}\left(t_{k+n}\right)\times 100\%$$

Where $C_{s}\left(t\right)$ denotes the binarized eye-closure signal, k is the window index, $t_{k}$ represents the time corresponding to the center of the 60 s window, $N_{60}$ the number of samples within that window and ⌊·⌋ the floor function.

The PERCLOS index was then computed on a second-by-second basis using a sliding 60-second window with a 1-second step size, resulting in a time-resolved drowsiness signal across the session, referred to as the PERCLOS signal.

Subsequently, the drowsigram was created by thresholding the PERCLOS signal according to the criteria proposed by Wierwille et al.^29^ with the addition of a fourth state, microsleep, to capture prolonged eye closures. The classification scheme is defined as follows:

(Equation 6) $$D_{s,k}=\left\{ \begin{array}{ll} Awake, & 0.00\leq \text{PERCLO}S_{s,k}<0.08\\ Likely\;Drowsy, & 0.08\leq \text{PERCLO}S_{s,k}<0.15\\ Drowsy, & \;0.15\leq \text{PERCLO}S_{s,k}<1.00\\ Microsleep, & Eyes\;continuously\;closed\;for\;more\;than\;5\;seconds \end{array}\right.$$

Where $D_{s,k}$ is the drowsiness level for the participant s in the $k^{th}$ 60 seconds’ window?

Finally, the drowsigram was resampled to 1 Hz to match the rs-fMRI repetition time parameter.

#### Calculation of the BOLD signal spectral features according to the “drowsigram”

We determined the frequency content of the extracted time series from all ROIs of each scan by computing a multitaper spectrogram (spectral resolution = 0.016 Hz, window length = 60 s, step = 1 s, Time Half Bandwidth Product = 2.5, Number of tapers = 4, NFFT = 64, detrend = linear30, where the window length used was chosen to match the data from PERCLOS. The spectrum examined was limited by two factors. Firstly, the TR of 1 second was insufficient to examine frequencies above 0.5 Hz. Secondly, the window length of the taper spectrogram further restricts the examination to 33 frequency bands within this 0-0.5 Hz range. We segregated the columns of all spectrograms by the states in the “drowsigram,” resulting in unique spectrograms, each representing the frequency content in a specific state. Thereafter, these spectrograms were averaged row-wise and exported for statistical analysis (see the following section). Subsequently, participant-wise averaging was performed to obtain global spectra representing the Power Spectral Density (PSD) in each of the 33 frequency bands and for each state in the “drowsigram” (see Graphical Abstract). Ultimately, we produced brain images (Figure 3) illustrating the PSD across the first 6 frequency bands [0-0.1Hz] which are indicative of the brain activity analyzed through BOLD during sleep.^16^

### Quantification and statistical analysis

The statistical analysis followed three steps after the verification of data distribution with the Shapiro-Wilk test. As a first step, we assessed the alignment between participants’ self-reported drowsiness states in the RSQ and the data obtained through the classification of PERCLOS index. This involved conducting a two-way analysis of variance (ANOVA) on the PERCLOS index while considering the different states in the “drowsigram” and the participants’ self-reported RSQ answers, as the factors. In the second step, we tested whether the “drowsigram” affected the PSD of BOLD oscillations. We opted to analyze the PSD of BOLD oscillations at 0.05 Hz (PSD0.05) as observation of changes related to the “drowsigram” peaked at 0.05 Hz. We then computed Generalized Linear Models (GLMs) for each of the 164 Region Of Interests (ROIs) in the AAL3 1 mm atlas, systematically examining the impact of different states of the “drowsigram” on the PSD0.05. We also conducted correlational analyses between PERCLOS and PSD0.05 for each participant across all 164 ROIs using linear regression. After this analysis in the ROIs, and as a third step, we extended our inquiry to evaluate the influence of the “drowsigram” on the PSD0.05 within established functional networks, as defined in the atlas of Thomas Yeo et al.^50^ This broader analysis employed a linear mixed model, with participants considered as random factors and the states in the “drowsigram” as well as functional networks treated as fixed factors. To ensure robust results, we applied a significance threshold of 0.05 to all our analyses, and a Bonferroni correction for multiple analyses and also for pairwise post-hoc comparisons. Statistical significance in the figures is indicated using asterisks as follows: ∗p < 0.05; ∗∗p < 0.01; ∗p < 0.001. All analyses were performed with SPSS 24.0® software (IBM; Armonk, NY, USA).
