# Supplementary material for: Dynamic functional MRI markers of drowsiness during sleep onset period
Source: iScience. 2025 Jul 10;28(8):113088. doi: 10.1016/j.isci.2025.113088 (PMC12312039; doi:10.1016/j.isci.2025.113088)
Supplement: Document S1. Figures S1–S9 and Tables S1 and S2 [file mmc1.pdf]

iScience, Volume *28*

Supplemental information

Dynamic functional MRI markers

# of drowsiness during sleep onset period

Ivan Igor Gaez, Elpidio Attoh-Mensah, Clément Nathou, Lydie Vincent, Marc Joliot, Luc Brun, Mikaël Naveau, and Olivier Etard

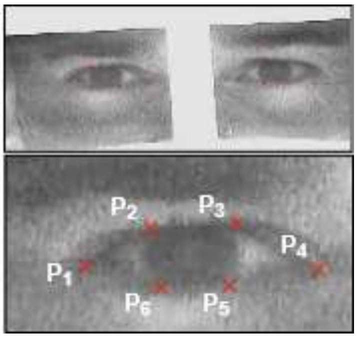

Figure S1. Eye landmarks annotation scheme. Related to STAR Methods. Landmarks were annotated on the left eye as follows: p_1 at the lateral canthus, p_2 at the outer intersection of the iris and the upper eyelid, p_3 at the inner intersection of the iris and the upper eyelid, p_4 at the medial canthus, p_5 at the inner intersection of the iris and the lower eyelid, and p_6 at the outer intersection of the iris and the lower eyelid. The same scheme was mirrored for the annotation of the right eye although not used.

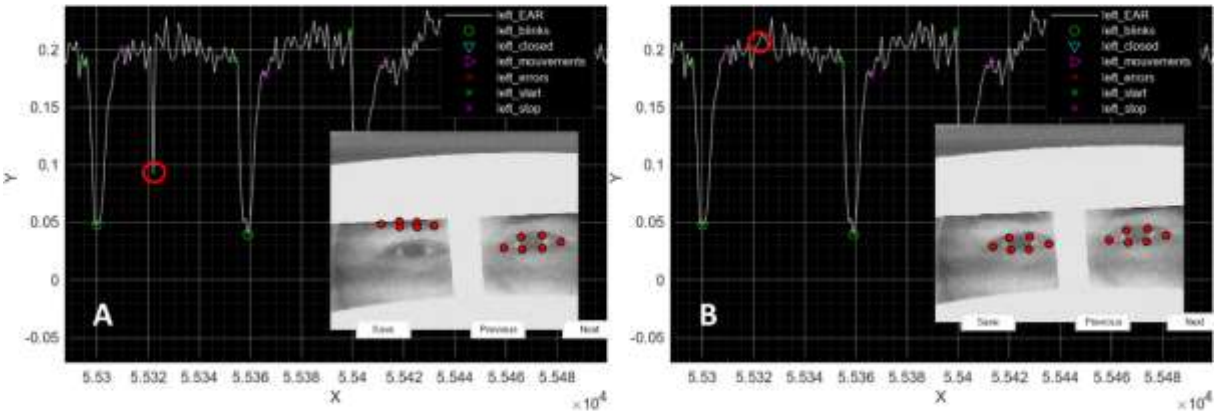

Figure S2. Two screenshots from the landmark correction user interface. Related to STAR Methods. In both panels, the X-axis represents the frame number and the Y-axis represents the Eye Aspect Ratio (EAR). A superimposed image of the participant’s face is shown in each screenshot. A) Depicts a case of false landmark detection, which leads to an artifact in the EAR signal (highlighted with an unfilled red circle). B) Shows the corrected landmark detection and its corresponding effect on the EAR signal (also highlighted with an unfilled red circle).

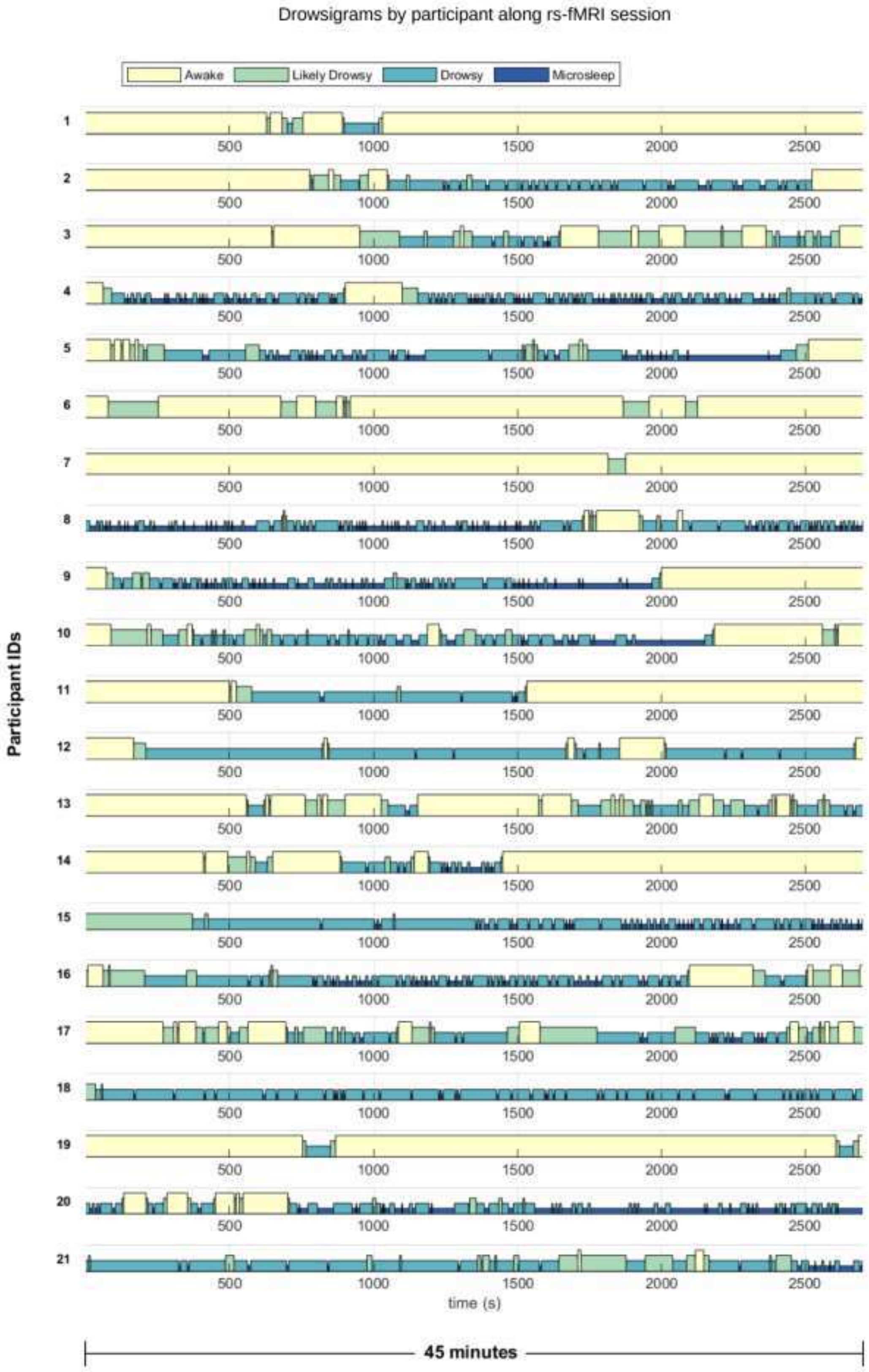

Figure S3. Drowsigram by participant along rs-fMRI session. Related to Figure 1. Time spent in each state of the drowsigram was depicted for the entire 45 minutes.

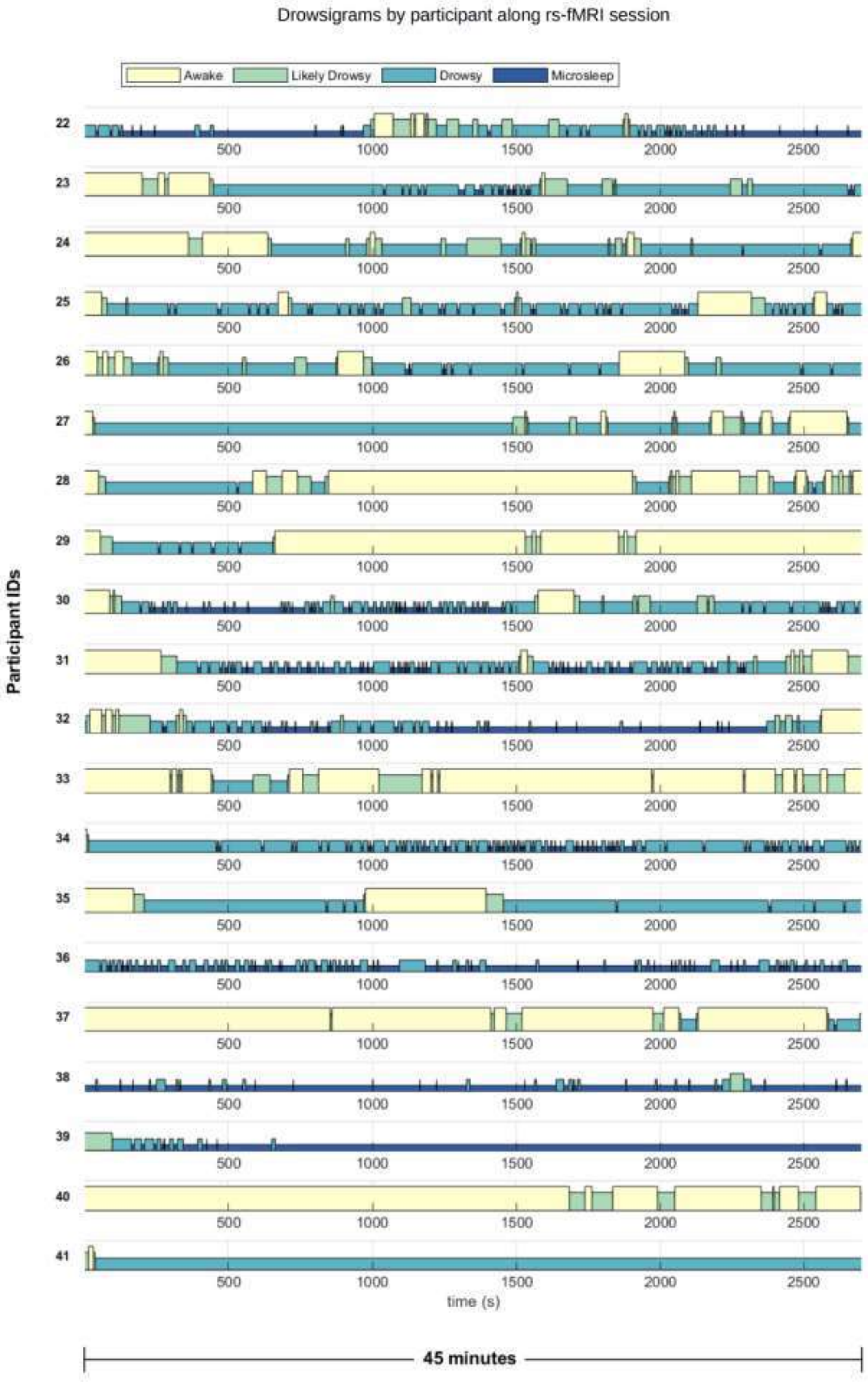

Figure S4. Drowsigram by participant along rs-fMRI session. Related to Figure 1. Time spent in each state of the drowsigram was depicted for the entire 45 minutes.

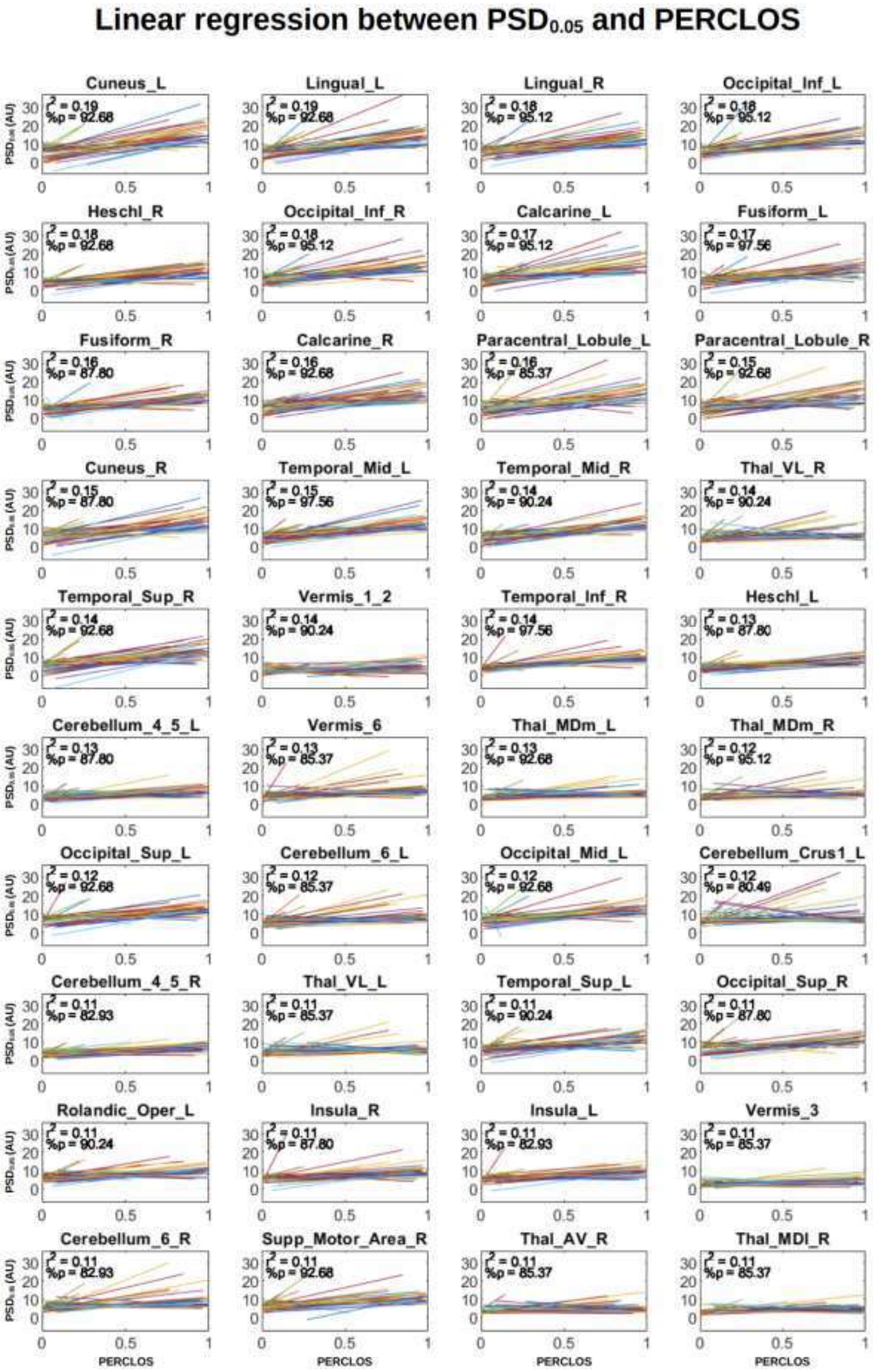

Figure S5. Linear regression between PSD at 0.05Hz and PERCLOS index. Related to Figure 2. The AAL3 regions have been sorted by R^2 from the largest to the smallest value. %p depicted on the plots corresponds to the percentage of subjects for which the pearson's p correlation is significative after correction by Bonferroni.

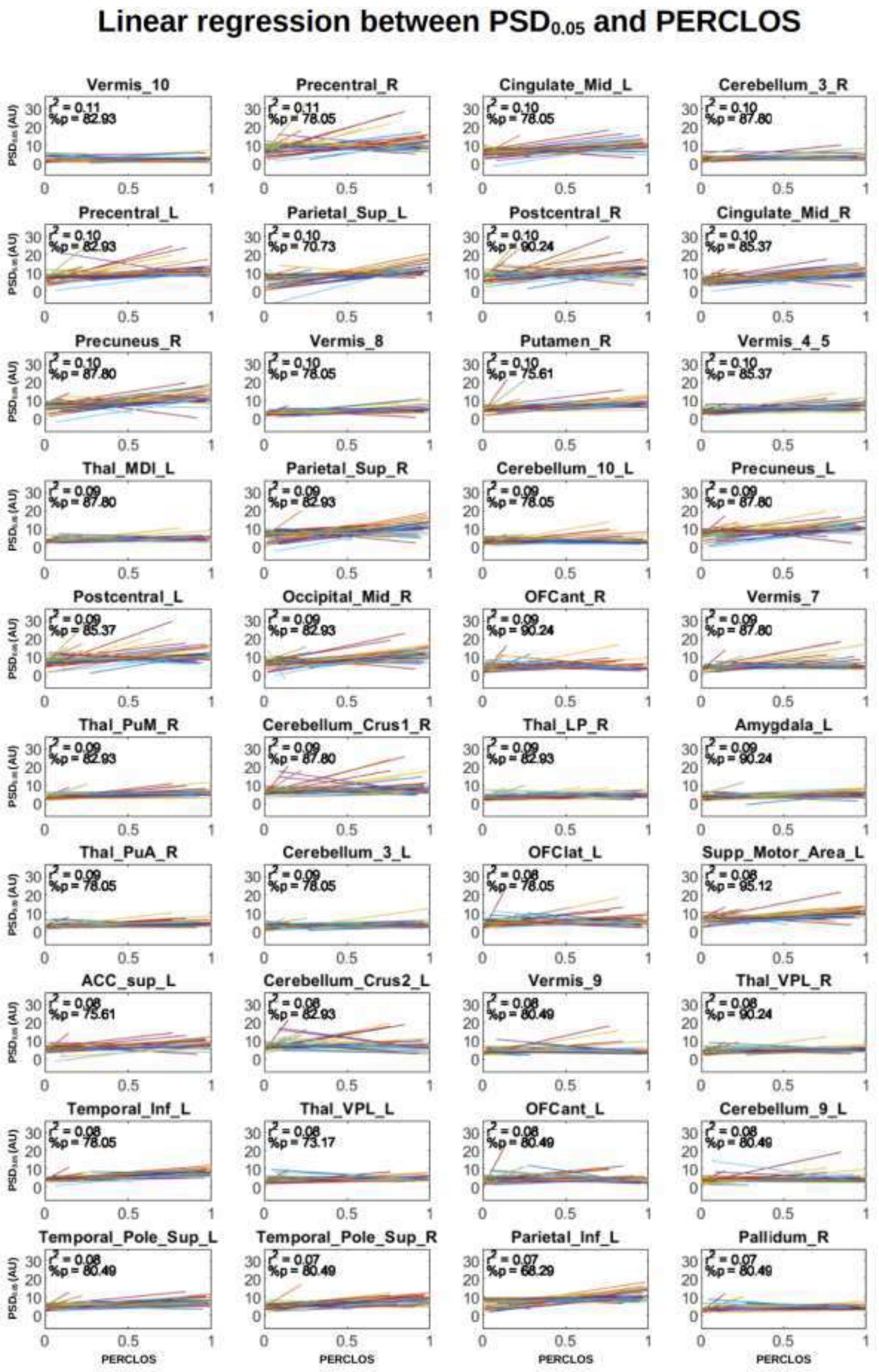

Figure S6. Linear regression between PSD at 0.05Hz and PERCLOS index. Related to Figure 2. The AAL3 regions have been sorted by R^2 from the largest to the smallest value. %p depicted on the plots corresponds to the percentage of subjects for which the pearson's p correlation is significative after correction by Bonferroni.

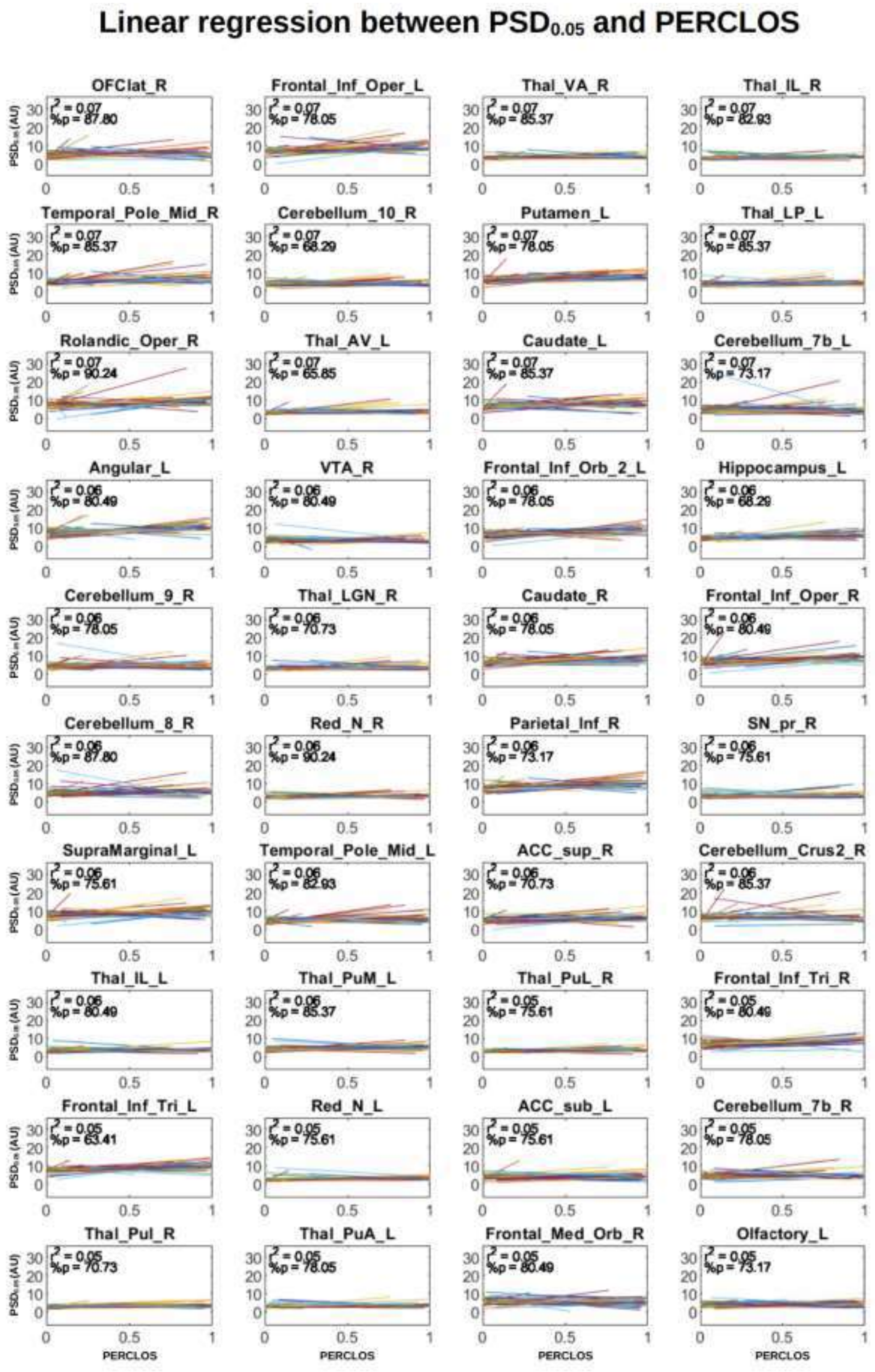

Figure S7. Linear regression between PSD at 0.05Hz and PERCLOS index. Related to Figure 2. The AAL3 regions have been sorted by R^2 from the largest to the smallest value. %p depicted on the plots corresponds to the percentage of subjects for which the pearson's p correlation is significative after correction by Bonferroni.

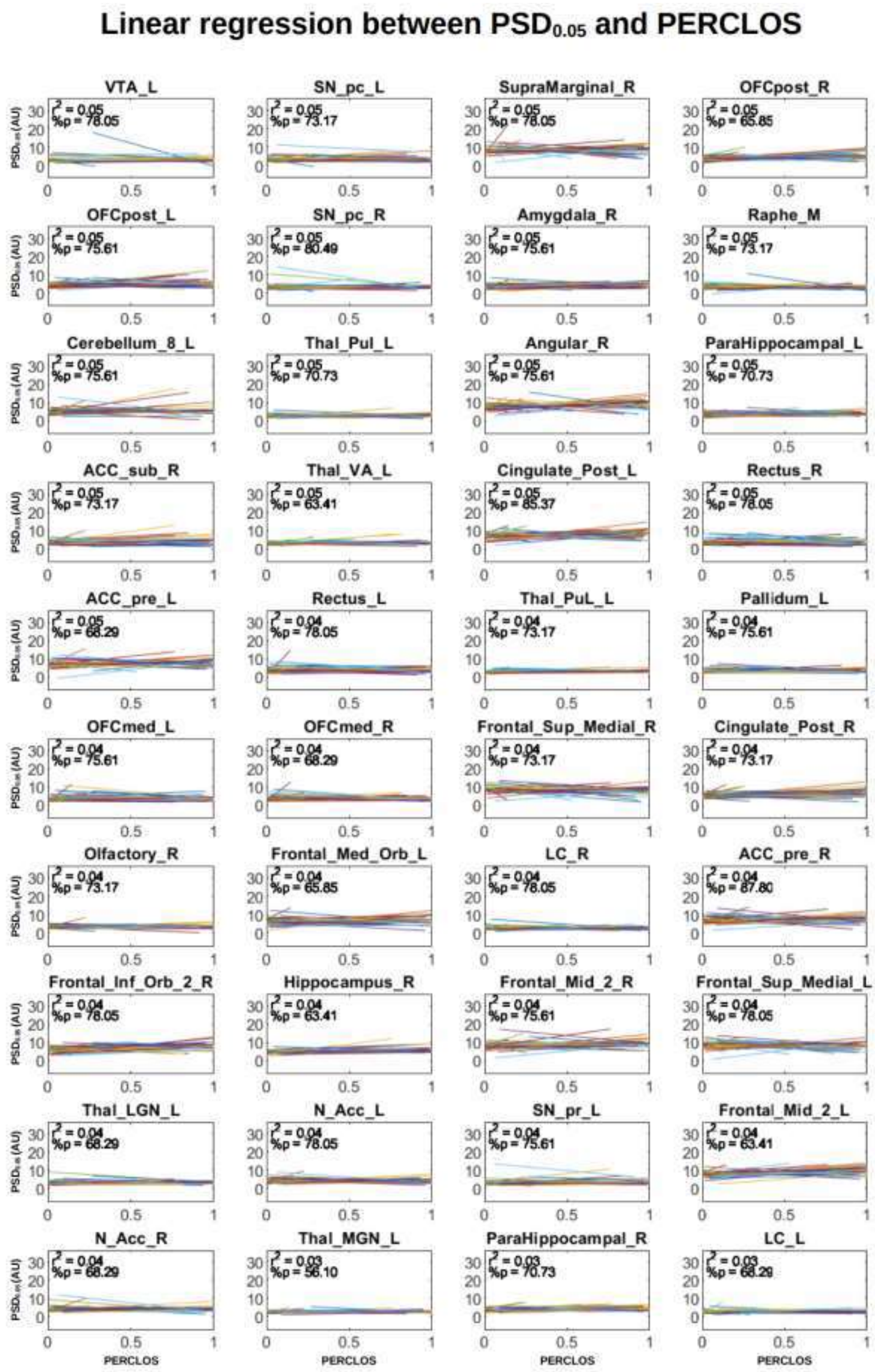

Figure S8. Linear regression between PSD at 0.05Hz and PERCLOS index. Related to Figure 2. The AAL3 regions have been sorted by R^2 from the largest to the smallest value. %p depicted on the plots corresponds to the percentage of subjects for which the pearson’s p correlation is significative after correction by Bonferroni.

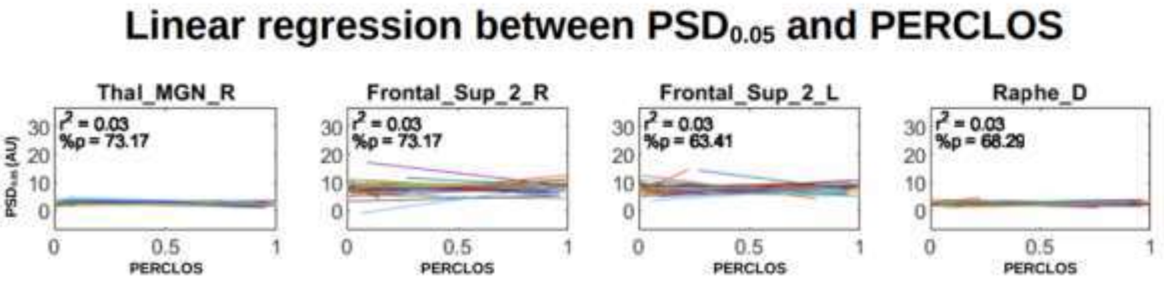

Figure S9. Linear regression between PSD at 0.05Hz and PERCLOS index. Related to Figure 2. The AAL3 regions have been sorted by R^2 from the largest to the smallest value. %p depicted on the plots corresponds to the percentage of subjects for which the pearson's p correlation is significative after correction by Bonferroni.

Table S1 Related to Table 1

Supplementary Table S1. Overview of statistically significant changes in $PSD_{0.05}$ across the drowsigram

	GEE[a]		Sleep vs Drowsy		Sleep vs Likely Drowsy		Likely Drowsy vs Drowsy		Sleep vs Awake		Drowsy vs Awake		Likely Drowsy vs Awake	
ROI[b]	p	Effect Size[c]	p	Effect Size[d]	p	Effect Size[d]	p	Effect Size[d]	p	Effect Size[d]	p	Effect Size[d]	p	Effect Size[d]
Precentral_L	0,00000001	44,68	0,02048	0,07988	0,00028	0,12733	0,01600	0,16304	0,00000	0,19196	0,00000	0,43117	0,00194	0,21913
Precentral_R	0,000001	31,807	0,14908	0,03891	0,01913	0,06257	0,15080	0,07035	0,00004	0,11739	0,00000	0,27547	0,00060	0,19427
Frontal_Sup_2_L	0,963	0,263												
Frontal_Sup_2_R	0,031	8,87												
Frontal_Mid_2_L	0,052	7,73												
Frontal_Mid_2_R	0,0001	15,908	0,55128	-0,04760	0,25974	0,08099	0,06598	0,13423	0,00835	0,22427	0,00027	0,35747	0,18281	0,10019
Frontal_Inf_Oper_L	0,000001	30,69	0,00050	0,24095	0,00013	0,25824	0,20807	0,09301	0,00000	0,39917	0,00062	0,38676	0,15181	0,10820
Frontal_Inf_Oper_R	0,000001	33,29	0,08485	0,13219	0,00565	0,14783	0,05512	0,09436	0,00000	0,39418	0,00004	0,46468	0,59995	0,02583
Frontal_Inf_Tri_L	0,00001	21,55	0,00239	0,22645	0,00002	0,31242	0,02172	0,17568	0,00004	0,31893	0,04310	0,17683	0,70793	0,02745
Frontal_Inf_Tri_R	0,006	12,6												
Frontal_Inf_Orb_2_L	0,000387	18,27												
Frontal_Inf_Orb_2_R	0,001	17,57												
Rolandic_Oper_L	1E-10	50,73	0,01100	0,17262	0,00019	0,24852	0,07985	0,12361	0,00000	0,47187	0,00000	0,45303	0,00543	0,20906
Rolandic_Oper_R	0,001	16,71												
Supp_Motor_Area_L	0,000001	31,6	0,00044	0,19361	0,00030	0,20148	0,45776	0,05321	0,00000	0,27182	0,01614	0,23840	0,22821	0,08773
Supp_Motor_Area_R	1E-12	59,48	0,00122	0,19285	0,00000	0,27675	0,03403	0,13906	0,00000	0,37931	0,00026	0,30004	0,22237	0,08384
Olfactory_L	0,131	5,62												
Olfactory_R	0,04	8,34												
Frontal_Sup_Medial_L	0,229	4,31												
Frontal_Sup_Medial_R	0,174	5,01												
Frontal_Med_Orb_L	0,107	6,09												
Frontal_Med_Orb_R	0,019	9,92												
Rectus_L	0,263	3,98												
Rectus_R	0,061	7,36												
OFCmed_L	0,005	12,92												
OFCmed_R	0,212	4,503												
OFCant_L	0,027	9,19												
OFCant_R	0,004	13,14												
OFCpost_L	0,017	10,28												
OFCpost_R	0,009	11,59												
OFClat_L	0,01	11,3												
OFClat_R	0,000272	19,01	0,98530	-0,00136	0,44617	0,05438	0,36333	0,07186	0,00844	0,20363	0,00147	0,30847	0,03744	0,17926
Insula_L	0,000000001	44,92	0,00325	0,24798	0,00737	0,16422	0,40195	0,04706	0,00000	0,51592	0,00002	0,52448	0,07763	0,10276
Insula_R	0,00000001	43,11	0,00379	0,20116	0,00398	0,14990	0,26199	0,05355	0,00000	0,39776	0,00011	0,42025	0,23326	0,05831
Cingulate_Mid_L	0,0000001	36,15	0,11955	0,11849	0,00000	0,45460	0,00012	0,31231	0,00088	0,19348	0,01900	0,12678	0,67506	0,02155
Cingulate_Mid_R	0,00000001	41,776	0,00977	0,21291	0,00000	0,49953	0,00003	0,38359	0,00000	0,36660	0,00105	0,24166	0,80017	0,01758
Cingulate_Post_L	0,76	1,14												
Cingulate_Post_R	0,874	0,69												
Hippocampus_L	0,001	16,62												
Hippocampus_R	0,002	15,28												
ParaHippocampal_L	0,443	2,68												
ParaHippocampal_R	0,000463	17,89												
Amygdala_L	0,004	13,15												
Amygdala_R	0,001	15,95												
Calcarine_L	1E-19	98,72	0,00055	0,13053	0,00000	0,25498	0,00000	0,31706	0,00000	0,29012	0,00000	0,48542	0,22679	0,07872
Calcarine_R	1E-19	111,41	0,00015	0,16973	0,00000	0,30373	0,00001	0,27776	0,00000	0,36782	0,00000	0,50816	0,18841	0,08210
Cuneus_L	0,000000001	48,06	0,00076	0,10408	0,00000	0,18070	0,00017	0,19643	0,00000	0,19629	0,00001	0,25113	0,41141	0,04899
Cuneus_R	0,000000001	48,84	0,00017	0,14537	0,00000	0,20637	0,00934	0,14480	0,00000	0,25089	0,00002	0,31022	0,22904	0,06875
Lingual_L	1E-15	77,02	0,02455	0,07839	0,00000	0,20506	0,00000	0,31816	0,00000	0,23031	0,00000	0,41264	0,22407	0,08610
Lingual_R	1E-19	97,84	0,00011	0,18911	0,00000	0,37048	0,00000	0,31637	0,00000	0,41990	0,00000	0,43410	0,30958	0,07213
Occipital_Sup_L	1E-11	58,52	0,00035	0,24165	0,00007	0,19024	0,07201	0,07735	0,00000	0,52729	0,00000	0,40167	0,12078	0,06793
Occipital_Sup_R	0,000001	32,03	0,01944	0,14892	0,00004	0,24700	0,01862	0,13290	0,00000	0,41545	0,00017	0,27684	0,35489	0,05394
Occipital_Mid_L	0,000001	33,9	0,02791	0,11024	0,00000	0,24601	0,00109	0,17884	0,00008	0,17515	0,00807	0,11154	0,98631	0,00069
Occipital_Mid_R	0,007	12,26												
Occipital_Inf_L	1E-12	61,74	0,01640	0,11131	0,00000	0,35978	0,00000	0,23486	0,00000	0,27497	0,00008	0,17737	0,96447	0,00196
Occipital_Inf_R	1E-19	87,01	0,00193	0,12929	0,00000	0,29144	0,00000	0,39872	0,00000	0,26068	0,00001	0,24528	0,87157	0,00803
Fusiform_L	1E-13	64,55	0,00817	0,13294	0,00000	0,26132	0,00013	0,23357	0,00000	0,37386	0,00000	0,60874	0,11228	0,09508
Fusiform_R	1E-10	53,23	0,00120	0,21803	0,00000	0,45921	0,00003	0,30128	0,00000	0,45869	0,00001	0,31014	0,50131	0,04326
Postcentral_L	0,0000001	39,57	0,16309	0,05008	0,04451	0,07144	0,38999	0,04540	0,00000	0,18299	0,00000	0,31262	0,00003	0,25372
Postcentral_R	0,000001	32,48	0,30360	0,02208	0,05743	0,03968	0,14021	0,06550	0,00046	0,07691	0,00000	0,23772	0,00034	0,20060
Parietal_Sup_L	0,000381	18,3												
Parietal_Sup_R	0,002	14,91												
Parietal_Inf_L	0,000000001	46,94	0,00001	0,30544	0,00000	0,43373	0,00087	0,27450	0,00000	0,38670	0,03340	0,18921	0,21455	0,09569
Parietal_Inf_R	0,0001	25,81	0,31882	0,05849	0,00174	0,18985	0,00138	0,26199	0,00045	0,21746	0,00003	0,48958	0,87763	0,01247
SupraMarginal_L	0,000333	18,56												
SupraMarginal_R	0,43	2,72												
Angular_L	0,029	9,04												
Angular_R	0,231	4,29												
Precuneus_L	0,000329	18,61												
Precuneus_R	0,00001	28,08	0,00020	0,18958	0,00001	0,23523	0,06918	0,12225	0,00000	0,28125	0,00347	0,26066	0,49048	0,04647
Paracentral_Lobule_L	1E-12	62,92	0,01500	0,10414	0,00000	0,22677	0,00473	0,13211	0,00000	0,34055	0,00000	0,26403	0,01337	0,14308
Paracentral_Lobule_R	1E-13	65,84	0,00127	0,13428	0,00000	0,21927	0,00440	0,16657	0,00000	0,30088	0,00000	0,37484	0,01052	0,16753
Caudate_L	0,001	15,99												
Caudate_R	1E-11	57,98	0,19410	0,11090	0,01646	0,19465	0,07537	0,15413	0,00000	0,50195	0,00000	0,87779	0,00325	0,26623
Putamen_L	0,000113	20,86	0,00306	0,31827	0,03224	0,12869	0,59612	0,02865	0,00012	0,32877	0,02335	0,17666	0,29159	0,06006
Putamen_R	0,00001	25,75	0,29108	0,08335	0,00900	0,15998	0,04157	0,11866	0,00001	0,39543	0,00005	0,39693	0,32536	0,05754
Pallidum_L	0,032	8,78												
Pallidum_R	0,06	7,41												
Heschl_L	1E-14	70,06	0,01621	0,17926	0,00000	0,38286	0,00033	0,31853	0,00000	0,54144	0,00000	0,65421	0,09424	0,15268
Heschl_R	1E-19	96,46	0,00098	0,20158	0,00000	0,38587	0,00012	0,31704	0,00000	0,48213	0,00000	0,54412	0,06726	0,17089
Temporal_Sup_L	1E-19	108,6	0,04819	0,10421	0,00107	0,16064	0,03090	0,10917	0,00000	0,39314	0,00000	0,66762	0,00169	0,16322
Temporal_Sup_R	1E-19	108,72	0,01715	0,08590	0,00001	0,16945	0,00103	0,15749	0,00000	0,26586	0,00000	0,46122	0,00390	0,14327

[a] Generalized Estimating Equations

[b] ROI labels of AAL3 atlas

[c] Wald's effect size

[d] Cohen's d effect size

The analyses correspond to Bonferroni-corrected pairwise comparisons after generalized linear models for the 164 Regions of Interest(ROI) in the AAL3 atlas (p<00005)

Table S2 Related to Table 1

Supplementary Table S2. Overview of statistically significant changes in $PSD_{0.05}$ across the drowsigram

	GEE[a]		Sleep vs Drowsy		Sleep vs Likely Drowsy		Likely Drowsy vs Drowsy		Sleep vs Awake		Drowsy vs Awake		Likely Drowsy vs Awake	
ROI[b]	p	Effect Size[c]	p	Effect Size[d]	p	Effect Size[d]	p	Effect Size[d]	p	Effect Size[d]	p	Effect Size[d]	p	Effect Size[d]
Temporal_Pole_Sup_L	0,00000001	43,44	0,033440	0,189145	0,000907	0,261247	0,060848	0,144837	0,000000	0,535836	0,000017	0,501396	0,095545	0,131338
Temporal_Pole_Sup_R	1E-12	62,29	0,079317	0,104111	0,000024	0,258541	0,001585	0,250685	0,000000	0,353472	0,000001	0,445510	0,028348	0,218930
Temporal_Mid_L	1E-15	74,15	0,050821	0,067840	0,000001	0,182491	0,000001	0,320312	0,000000	0,216415	0,000000	0,578449	0,161748	0,090781
Temporal_Mid_R	1E-15	77,74	0,003682	0,135863	0,000000	0,328285	0,000000	0,415423	0,000000	0,366430	0,000000	0,592051	0,454519	0,054403
Temporal_Pole_Mid_L	0,000467	17,87												
Temporal_Pole_Mid_R	0,000174	19,94	0,631185	0,033529	0,001846	0,221272	0,000570	0,321824	0,000813	0,246619	0,000095	0,426854	0,863067	0,016371
Temporal_Inf_L	1E-15	76,88	0,009877	0,230857	0,000000	0,544842	0,000006	0,540479	0,000000	0,602294	0,000000	0,690222	0,690400	0,045748
Temporal_Inf_R	0,000000001	46,27	0,001273	0,242601	0,000000	0,340454	0,001397	0,196294	0,000000	0,462685	0,000008	0,318850	0,276734	0,062416
Cerebellum_Crus1_L	1E-12	59,4	0,297197	0,032022	0,014302	0,073157	0,049637	0,096157	0,000010	0,137465	0,000000	0,280410	0,000047	0,280513
Cerebellum_Crus1_R	0,0000001	37,74	0,094905	0,052122	0,005118	0,088343	0,056018	0,106689	0,000040	0,131720	0,000003	0,336107	0,021197	0,137597
Cerebellum_Crus2_L	0,0000001	39,64	0,764421	0,012126	0,737267	0,013524	0,934137	0,004494	0,002278	0,125181	0,000008	0,369222	0,000396	0,204298
Cerebellum_Crus2_R	0,027	9,16												
Cerebellum_3_L	0,000433	18,03												
Cerebellum_3_R	0,000001	35,07	0,074086	0,178938	0,000186	0,379350	0,017246	0,312881	0,000728	0,356537	0,039858	0,270178	0,861574	0,025398
Cerebellum_4_5_L	1E-14	68,59	0,000282	0,338567	0,000000	0,481514	0,009671	0,254736	0,000000	0,707877	0,000002	0,600737	0,051682	0,193453
Cerebellum_4_5_R	1E-12	60,32	0,001598	0,265679	0,000002	0,396896	0,009698	0,227630	0,000000	0,580293	0,000009	0,544401	0,234561	0,106440
Cerebellum_6_L	0,000000001	47,73	0,033715	0,081858	0,000002	0,187308	0,000059	0,260418	0,000001	0,200553	0,000010	0,303683	0,536424	0,051257
Cerebellum_6_R	0,00001	29,25	0,044313	0,071170	0,000100	0,138699	0,000328	0,295742	0,000008	0,169937	0,000001	0,432116	0,074254	0,152357
Cerebellum_7b_L	0,578	1,974												
Cerebellum_7b_R	0,000489	17,76												
Cerebellum_8_L	0,083	6,68												
Cerebellum_8_R	0,037	8,46												
Cerebellum_9_L	0,036	8,52												
Cerebellum_9_R	0,04	8,33												
Cerebellum_10_L	0,02	9,83												
Cerebellum_10_R	0,029	8,99												
Vermis_1_2	0,283	3,81												
Vermis_3	0,000325	18,63												
Vermis_4_5	1E-12	63,06	0,001960	0,336094	0,000000	0,600535	0,000273	0,397639	0,000000	0,778239	0,000001	0,626086	0,299906	0,108144
Vermis_6	0,0000001	37,44	0,022359	0,091419	0,001213	0,135778	0,047613	0,113472	0,000003	0,202290	0,000000	0,556875	0,077126	0,102488
Vermis_7	0,0001	24,83	0,158815	0,075094	0,008017	0,143610	0,037316	0,194912	0,000105	0,217490	0,000007	0,547836	0,036939	0,205629
Vermis_8	0,000000001	46,72	0,001269	0,366370	0,000001	0,572206	0,004694	0,361019	0,000000	0,774932	0,000008	0,738164	0,208275	0,161934
Vermis_9	0,000134	20,48	0,746960	0,027363	0,120753	0,130062	0,128570	0,154261	0,000503	0,312763	0,000061	0,492436	0,015893	0,268700
Vermis_10	0,481	2,168												
Thal_AV_L	0,000126	20,61	0,665105	0,061341	0,127998	0,160244	0,179837	0,134633	0,000083	0,610672	0,000044	0,731141	0,108089	0,163992
Thal_AV_R	0,00000001	40,96	0,543824	0,062739	0,044135	0,199295	0,075526	0,194109	0,000002	0,543535	0,000000	0,818618	0,002442	0,350018
Thal_LP_L	0,000001	33,78	0,993416	0,001258	0,986403	-0,000791	0,982737	-0,000935	0,000009	0,774329	0,000002	0,909524	0,093242	0,074084
Thal_LP_R	1E-10	50,31	0,364488	0,079360	0,077262	0,134726	0,178539	0,102106	0,000003	0,441796	0,000000	0,932127	0,017084	0,184542
Thal_VA_L	0,000449	17,95												
Thal_VA_R	0,00001	30,55	0,277107	0,127054	0,001238	0,388856	0,000600	0,709502	0,000050	0,516518	0,000001	1,162763	0,121832	0,309573
Thal_VL_L	0,001	17,35												
Thal_VL_R	0,0001	24,44	0,317110	0,057927	0,018274	0,137454	0,046996	0,150823	0,000017	0,273519	0,000001	0,463150	0,008678	0,200509
Thal_VPL_L	0,001	17,31												
Thal_VPL_R	0,0000001	35,84	0,085920	0,190121	0,002362	0,305303	0,063393	0,185769	0,000001	0,590706	0,000038	0,531751	0,059402	0,190029
Thal_IL_L	0,016	10,33												
Thal_IL_R	0,015	10,48												
Thal_MDm_L	0,0000001	38,05	0,119007	0,145862	0,000026	0,395321	0,000351	0,370645	0,000000	0,512512	0,000001	0,528920	0,138265	0,137207
Thal_MDm_R	0,00000001	43,98	0,139811	0,119775	0,000114	0,321256	0,001472	0,324041	0,000000	0,495952	0,000000	0,661385	0,022121	0,240668
Thal_MDl_L	0,0001	21,48	0,091388	0,192695	0,001023	0,382980	0,012643	0,378293	0,000050	0,499830	0,000192	0,582498	0,201221	0,174793
Thal_MDl_R	0,02	9,76												
Thal_LGN_L	0,006	12,5												
Thal_LGN_R	0,187	4,8												
Thal_MGN_L	0,006	12,3												
Thal_MGN_R	0,092	6,43												
Thal_PuI_L	0,011	11,1												
Thal_PuI_R	0,002	14,55												
Thal_PuM_L	0,000001	31,32	0,297209	0,150126	0,024273	0,293142	0,149196	0,181501	0,001746	0,349651	0,009887	0,270898	0,182420	0,133697
Thal_PuM_R	0,021	9,75												
Thal_PuA_L	0,0000001	37,75	0,742000	0,055955	0,245430	0,172895	0,342744	0,138080	0,010789	0,422075	0,013924	0,404139	0,178443	0,192059
Thal_PuA_R	0,001	15,48												
Thal_PuL_L	0,003	14,19												
Thal_PuL_R	0,002	14,79												
ACC_sub_L	0,000101	21,09	0,283350	0,083081	0,040761	0,155973	0,276749	0,108857	0,003588	0,233809	0,022587	0,241036	0,134810	0,193094
ACC_sub_R	0,054	7,62												
ACC_pre_L	0,016	10,31												
ACC_pre_R	0,0001	24,28	0,180277	-0,129453	0,679355	-0,030451	0,498974	0,048008	0,185170	0,122187	0,003802	0,290501	0,109946	0,115868
ACC_sup_L	0,057	7,51												
ACC_sup_R	0,417	2,84												
N_Acc_L	0,442	2,69												
N_Acc_R	0,224	4,36												
VTA_L	0,902	0,57												
VTA_R	0,017	10,15												
SN_pc_L	0,858	0,76												
SN_pc_R	0,456	2,61												
SN_pr_L	0,135	5,56												
SN_pr_R	0,76	1,16												
Red_N_L	0,031	8,87												
Red_N_R	0,0444	2,67												
LC_L	0,967	0,26												
LC_R	0,031	8,9												
Raphe_D	0,83	0,86												
Raphe_M	0,112	5,99												

[a] Generalized Estimating Equations
[b] ROI labels of AAL3 atlas
[c] Wald's effect size
[d] Cohen's d effect size
The analyses correspond to Bonferroni-corrected pairwise comparisons after generalized linear models for the 164 Regions of Interest(ROI) in the AAL3 atlas (p<00005)
